# Myeloid cells in COVID-19 microenvironment

**DOI:** 10.1038/s41392-021-00792-0

**Published:** 2021-10-27

**Authors:** Guohui Qin, Shasha Liu, Li Yang, Weina Yu, Yi Zhang

**Affiliations:** 1 Biotherapy Center and Cancer Center, The First Affiliated Hospital of Zhengzhou University, Zhengzhou, 450052 China; 2 School of Life Sciences, Zhengzhou University, Zhengzhou, 450001 China; 3 Henan Key Laboratory for Tumor Immunology and Biotherapy, Zhengzhou, Henan 450052 China; 4 State Key Laboratory of Esophageal Cancer Prevention & Treatment, Zhengzhou University, Zhengzhou, 450052 China

**Keywords:** Infectious diseases, Immunotherapy, Infectious diseases

## Abstract

Varying differentiation of myeloid cells is common in tumors, inflammation, autoimmune diseases, and metabolic diseases. The release of cytokines from myeloid cells is an important driving factor that leads to severe COVID-19 cases and subsequent death. This review briefly summarizes the results of single-cell sequencing of peripheral blood, lung tissue, and cerebrospinal fluid of COVID-19 patients and describes the differentiation trajectory of myeloid cells in patients. Moreover, we describe the function and mechanism of abnormal differentiation of myeloid cells to promote disease progression. Targeting myeloid cell-derived cytokines or checkpoints is essential in developing a combined therapeutic strategy for patients with severe COVID-19.

## Introduction

As of 11 October 2021, there are 237 million confirmed coronavirus disease 2019 (COVID-19) cases, with more than 4 million deaths worldwide. Severe acute respiratory syndrome coronavirus 2 (SARS-CoV-2) transmission methods include aerosol mediation, respiratory droplets, or direct contact with contaminated surfaces. The typical symptoms of patients with COVID-19 are fever, cough, muscle pain, increased breathing rate, and pneumonia. Severe patients with COVID-19 infection also develop acute respiratory distress syndrome.

Binding to ACE2 receptor on the surface of epithelial cells to cause alveolar injury is the first step leading to acute respiratory distress syndrome by SARS-CoV-2. Successive studies have shown that immunopathology is significant to the progression of COVID-19, especially the emergence of severe cases. Among them, lymphopenia and “cytokine storm” are considered to be the main mechanisms resulting in severe COVID-19.^1^ A number of clinical investigations showed that whether in the peripheral circulation or alveolar lavage fluid, myeloid cells such as monocytes and neutrophils increased, mainly resulting in high levels of inflammatory factors such as interleukin 10 (IL-10) and IL-6. On the contrary, T cells and B cells decreased significantly.^2^

Under physiological homeostasis conditions, granulocyte-macrophage progenitors (GMPs) derived from common myeloid progenitors can be divided into two differentiation directions: myeloblasts and monocyte/macrophage precursors.^3^ Among these, myeloblasts differentiate into promyelocytic cells and pre-neutrophils in the bone marrow and then migrate to the peripheral blood to differentiate into mature neutrophils. Classical neutrophils exert a pathological role in regulating inflammation progression. Pro-monocytes differentiate into immature monocytes and then generate monocytes, which migrate into the peripheral blood and infected tissues (terminally differentiated macrophages and dendritic cells), and contribute to the process of pathogen elimination. However, inflammatory diseases, malignant tumors, and other disorders induce the abnormal accumulation of non-classical monocytes and neutrophils in the bone marrow. This pathologically activated state leads to loss of clearing the invading pathogens and inhibits adaptive immune response, resulting in disease progression.

The application of single-cell sequencing technology in tumors and COVID-19 has clarified the heterogeneity of differentiation trajectory and functions of immune cells. Moreover, the results in tumors have demonstrated that myeloid cells presented in various subpopulations and targets. In patients with triple-negative breast cancer, myeloid cells are transformed from macrophage-enriched type to a neutrophil-enriched type, which mediates the gradual formation of resistance to immune checkpoint blocking therapy.^4^ Upon monitoring the differentiation of monocytes into macrophages in tumor tissues of patients with metastatic lung adenocarcinoma, it has been determined that these macrophages lose their pro-inflammatory properties and acquire anti-inflammatory characteristics.^5^ These anti-inflammatory macrophages lose the ability to present antigens, leading to an immunosuppressive tumor microenvironment. In hepatocellular carcinoma, myeloid-derived suppressive cell (MDSC)-like cells have been identified, with upregulation of S100A family genes and FCN1 and downregulation of human leukocyte antigen (HLA)-related genes.^6^ Significant transcription factors or functional molecules are being resolved, leading to rapid progress in combined immunotherapy strategies targeting at myeloid cells. Therefore, the research strategies and analysis methods of single-cell sequencing in tumors can be maturely applied to the research of COVID-19, which support for the exploration of the differentiation trajectory, function, and significant targets of myeloid cells in the COVID-19 microenvironment.

In COVID-19 patients, when myeloid cells initially perceive the infection of “SARS-CoV-2,” the cells activated rapidly to eliminate pathogens through direct phagocytosis or indirect methods. Moreover, the abnormal “cytokine storm” or blood clotting caused by another myeloid cell subset is significant for the poor prognosis of severe COVID-19 patients. Therefore, clarifying the abnormal differentiation trajectory of myeloid cells and critical transcription factors in COVID-19 patients provides a strong foundation for developing treatment strategies and reducing the mortality of critically ill patients. This review analyzed the abnormal differentiation and pathological effects of myeloid cells in patients infected with SARS-CoV-2. It explored novel strategies targeting myeloid cells to improve the prognosis of severe patients.

## Myeloid cell differentiation in COVID-19

**Fig. 1:**
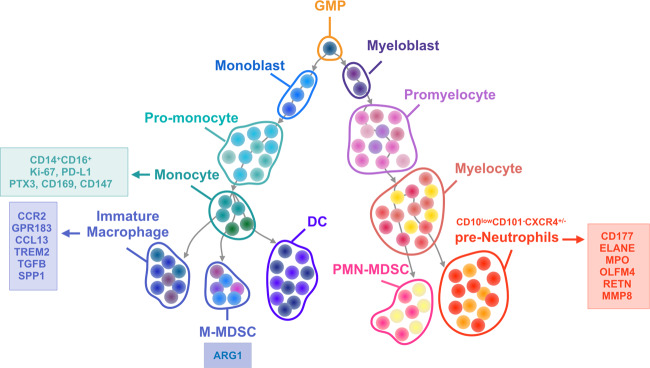
The differentiation trajectory of myeloid cells in COVID-19 microenvironment derived from the results of single-cell sequencing analysis. GMP differentiates into myeloblasts and monoblasts, and monoblasts differentiate into pro-monocytes and monocytes in the bone marrow. With COVID-19 infection, monocytes differentiate into macrophages and DCs in infected lung tissues and M-MDSCs in circulation. The expression of biomarkers such as Ki-67, PD-L1, PTX3, CD169, and CD147 was increased in monocytes, whereas monocytes mainly presented the CD14^+^CD16^+^ phenotype in severe COVID-19 patients. PMN-MDSC and pre-Neutrophil differentiation progresses through myeloblasts and myelocytes. In severe COVID-19 patients, pre-neutrophils such as CD10^low^CD101^−^CXCR4^+/−^ subsets, highly expressed CD177, ELANE, MPO, OLFM4, RETN, and MMP8

Single-cell sequencing has become a key technical method to describe the differentiation trajectory of myeloid cells in patients with COVID-19. Macrophages and neutrophils accumulate in large numbers, as the most active subgroup in bronchoalveolar lavage fluid, and are also identified as significant characteristics of severe COVID-19 patients (Fig. 1). In addition, the inflammatory factors such as tumor necrosis factor (TNF), IL-10, IL-6, or CCL3 and chemokines such as CXCL2, CCL8, or CCL20 have been investigated with higher levels through single-cell sequence. Moreover, single-cell sequencing demonstrated that macrophages with high expression of FCN1 and SPP1 have become the most subgroup in the alveolar lavage fluid of severely ill patients. In circulation, neutrophils were increased, but the proportion of CD14^+^ monocytes is lower in severe patients than in mild patients, and COX-2 secretion decreased.^7^ These preliminary findings of single-cell sequencing support that targeting myeloid cells from the bone marrow would be considered as potential treatment for COVID-19 infection.

The abnormal differentiation of myeloid cells and the increased production of cytokines leading to impaired adaptive immune response are the initiating factors for severe cases of COVID-19.^8,9^ The analysis of single-cell sequencing demonstrated that the inflammatory monocytes of myeloid cells persisted in the various stages of COVID-19 progression, namely the pre-severe, severe, late, and mild recovery phases.^10^ Therefore, these myeloid cell subsets have a continuous response to SARS-CoV-2 infection. A study from the United Kingdom analyzed the characteristics of immune cells derived from the peripheral blood of patients with COVID-19, showing that immune response activity is correlated with the ratio of neutrophils to T cells, the increase of IL-6, IP-10, or MCP-1 in serum, and changes in the characteristics of CD14^+^ monocytes.^7^ This study supports the hypothesis that myeloid cells play a vital role in the early stages of SARS-CoV-2 infection and the abnormal differentiation promotes the progression of severe COVID-19. The combined analysis of single-cell RNA and proteomics of immune cell subsets in the peripheral blood indicated that HLA-DR^hi^CD11c^hi^ inflammatory monocytes and interferon (IFN) signaling pathways were significantly increased in mild patients. However, in patients with severe cases of COVID-19, the accumulation of neutrophil precursor cells increased dramatically and their phenotypes and functions are similar to those of polymorphonuclear MDSCs (PMN-MDSCs).^11^ PMN-MDSCs have presented immunosuppressive functions in the tumor tissues and peripheral blood of patients with malignant tumors.^12^ This myeloid cell subset reduces the efficacy of immunotherapies and promotes tumor progression via cytokine secretion or cell–cell contact.^13^ However, it is still obscure whether these immunosuppressive cells are abundantly present in patients’ circulation system and infected lung tissues. This review systematically analyzed the abnormal differentiation state and function of myeloid cells from circulation to infected tissues, and verified the key molecules that control abnormal differentiation.

### Monocytes and macrophages

Monocytes are innate immune cells that eliminate pathogens and promote tissue repair by producing inflammatory factors.^14^ However, the abnormal differentiation and function of monocytes play a crucial role in promoting inflammation caused by pathogens, leading to severe cases of COVID-19. The autopsy results of patients who died due to COVID-19 showed that many monocytes had infiltrated the lymph nodes, kidneys, lungs, spleen, and other organs, whereas T cells were severely lacking.^15^ This indicated that abnormal changes in the ratio of monocytes to T cells are a vital driving factor leading to the death of severe COVID-19 patients.

Monocytes in the circulatory system migrate to tissues, to further polarize into macrophages or dendritic cells, resulting in an anti-pathogenic response.^16^ The progress of single-cell transcriptome technology has led to the gradual discovery of additional monocyte subtypes.^17^ Monocytes have been divided into three subgroups: classic CD14^+^CD16^−^, intermediate CD14^+^CD16^+^, and another group is CD14^dim^CD16^+^ cell populations. In SARS-CoV-2 patients, the proportion of intermediate CD14^+^CD16^+^-type monocytes significantly increased; it was higher in severe cases, whereas CD14^dim^CD16^+^ monocytes were lacking.^18,19^ These monocytes express high levels of CD80 and CD206, and secrete elevated levels of IL-6, TNF-α, and S100A8/A9, which are significantly positively correlated with disease progression.^18,20,21^ In another study including 69 patients with COVID-19 (14 patients with asymptomatic infections, 39 with non-severe illnesses, and 16 with severe conditions) and 21 healthy volunteers, immune cell investigation showed that the expression of CD4 in monocytes in the peripheral blood of severe patients decreased significantly, whereas the percentage of CD14^+^CD4^−^ monocytes increased. It has been proposed that CD4^−^ monocytes may be independent predictors of COVID-19 progression.^22^ In addition, the results of the horizontal analysis indicated that the expression of COX-2 was reduced in CD14^+^ monocytes of severely ill individuals. However, Ki-67 expression was remarkably increased; therefore, the proliferation ability of monocytes in infected patients was significantly enhanced.^7^ In addition, the function of mature monocytes was impaired and a large number of immature monocytes accumulated in severely infected patients. Moreover, the level of immune checkpoints, such as PD-L1, was increased significantly in impaired monocytes^19^.

In addition to the peripheral blood, abnormal accumulation and function of monocytes in alveolar lavage fluid can better reflect disease progression. A study investigated the differences in the differentiation trajectories of immune cell subsets in bronchoalveolar lavage fluid from 5 mild COVID-19 patients and 26 severe cases. The movement trajectory of monocytes to macrophages showed that chronic inflammation-related monocytes were abundant in patients with COVID-19. In contrast, alveolar macrophages lack the characteristics of anti-inflammatory and antigen presentation.^23^ The massive accumulation of mononuclear phagocytes (MNPs) has been identified as the critical factor in promoting acute inflammation and pulmonary interstitial fibrosis. Single-cell sequencing of alveolar lavage fluid showed that MNPs accounted for 80% of severely infected patients, whereas they were downregulated in patients with mild infections and healthy individuals, respectively.^15^ Another study analyzed the aggregation of immune cell subsets in the alveolar lavage fluid of six severely ill patients, three severely infected patients, and three healthy controls. The proportion of macrophages and neutrophils was higher in the alveolar lavage fluid of severe patients. The proportions of myeloid dendritic cells, platelet-like dendritic cells, and T cells were significantly lowered as the disease progressed.^2^ Analysis of macrophage subpopulations showed that the third group had M2-like macrophages with high expression of CCL13, GPR183, TGFB1, SPP1, and the pro-fibrotic genes TREM2, indicating that the M2-like macrophages accumulated in the alveolar lavage fluid promote fibrosis. Among these, fibrin exudation and dysfunction of alveolar macrophage accumulation are characteristic abnormalities. Thus, alveolar macrophages infected with SARS-CoV-2 are the driving factors of the “cytokine storm,” which resulting in the damage of lung tissues and multiple organ failure.

In contrast to the peripheral blood, analysis of the cerebrospinal fluid obtained from patients with the central nervous system infection revealed that monocyte subpopulations were abnormally differentiated earlier than other immune cells. Single-cell sequencing results showed that a group of monocytes that lost pan-monocyte markers (CD14/LYZ/CD68) and microglia or marginal-associated macrophage markers (CX3CR1/LYVE1/APOE/TREM2) were aggregated in the cerebrospinal fluid and this group was similar to the developing macrophages, with the phenotype of antigen presentation.^24^

### Increased neutrophils

Increased neutrophils are essential for inducing innate immune responses. They arrive at the infection site first through chemokine recruitment.^25^ This group of innate immune cells has a shorter life span after leaving the bone marrow. Nevertheless, these cell subsets exhibit anti-infection effects by cytokine release or phagocytosis in a short time. Increased neutrophils can also regulate adaptive immune responses by reducing the migration of T cells. In a study of influenza A virus, neutrophils showed the most effective antigen presentation cell activity to activate CD8^+^ T cells.^26^ The neutrophil-to-lymphocyte ratio (NLR) is positively associated with the development of inflammation in patients with COVID-19, i.e., in severely infected patients, the number of lymphocytes decreases and neutrophils become abundant, resulting in abnormally increased NLR.^27^ The results of the retrospective analysis found that for every unit increase in NLR, the mortality rate of in-patients increased by 8%.^28^ Furthermore, another retrospective analysis showed that an NLR > 13 was significantly positively correlated with mortality.^29^ Therefore, NLR may be an independent risk factor for patients with COVID-19 in-hospital death and the assessment of NLR may provide an insight to identify high-risk groups for COVID-19.

Probing the characteristic surface markers of abnormal increased neutrophils will help in the development of targeted treatment strategies. Elevated chemokines such as CXCL1, CXCL2, CXCL5, and CXCL8 recruit neutrophils by binding the chemokine receptor CXCR2 to the tumor microenvironment.^25,30^ The majority of clinical evidence suggests that NLR and neutrophilia are independent prognostic markers for many types of cancer. Increasing numbers of circulating neutrophils have been found in various cancer types.^31^ In 2009, Fridlender et al.^32^ first demonstrated that increased neutrophils have two polarization states, N1 and N2, which play different roles in tumor progression in mice model bearing subcutaneous mesothelioma tumors with the treatment of transforming growth factor (TGF)-β inhibitor. N1 mainly exerts an anti-tumorigenic role, whereas N2 has a pro-tumorigenic role. In the tumor microenvironment, TGF-β and granulocyte colony-stimulating factor (G-CSF) polarize neutrophils to the N2 phenotype and neutrophil depletion induces a decrease in tumor growth in mouse models.^33,34^ A single-cell sequencing study demonstrated that specific surface markers of neutrophils such as CD177 were highly expressed in critically ill patients.^35^ In addition, the expression of immunosuppressive molecules ARG1 and PD-L1 also increased significantly in neutrophils from severely infected patients. Neutrophil subgroup analysis shows that except for a large number of pro-inflammatory neutrophil aggregation, no mature CD10^low^CD101^−^CXCR4^+/−^ neutrophils accumulate in the peripheral blood and lung tissues to exert immunosuppressive function; this suggests the occurrence of bone marrow emergency hematopoiesis^20^ (Fig. 1). These cell subpopulations can be used as critical indicators for predicting the event of severe illnesses.

### Myeloid-derived suppressor cells

Longitudinal analysis of immune cell subsets derived from the peripheral blood of severe and mild patients from the early stage of symptom onset to the recovery stage, it was concluded that MDSCs accounted for most of the immune cells in severely infected patients. In contrast, the proportion of MDSCs in patients with mild disease was 25%.^36,37^ During recovery, the proportion of MDSCs gradually decreased as the secretion level of TGF-β was significantly inhibited. As an immature group of heterogeneous cell populations with immunosuppressive function, MDSCs are divided into M-MDSCs and PMN-MDSCs according to the differences in cell morphology and surface markers.^12,38,39^ In the study of malignant tumors, MDSCs are an independent prognostic biomarker for patients.^40–42^ By targeting CXCR2, LXR, PI3Kγ, and other checkpoints, reducing MDSC aggregation and inhibiting MDSC function can significantly improve the prognosis of patients with malignant tumors and restore the therapeutic effect of immune checkpoint inhibitors or Chimeric antigen receptor (CAR)-T cells.^43–45^ In COVID-19, M-MDSCs were identified to be remarkably increased in the peripheral blood; this proportion has been recommended as the biomarker to predict the progression of the disease.^37^ Similar to tumors, M-MDSCs derived from patients infected with COVID-19 inhibit T-cell proliferation as IFN-γ secretion depends on ARG1 expression and activity.

## Accumulation of abnormal myeloid cells

Abnormal differentiation and recruitment mechanisms are the main reasons for the significant accumulation of abnormal myeloid cells in circulation and lung tissues infected by COVID-19. This section summarizes the pivotal transcription factors that induce the abnormal differentiation of monocytes and neutrophils. We also describe the migration mechanism of these two subgroups of myeloid cells in the peripheral blood and various infected organs. This provides a new option for blocking the increase of abnormal myeloid cells.

### Transcription factors

PTX3 in bone marrow-derived monocytes is an integral part of innate humoral immunity and participates in eliminating pathogens.^46^ Plasma analysis showed that the concentration of PTX3 was significantly increased in 96 patients with COVID-19 pneumonia and PTX3 was more suitable for predicting a 28-day mortality than other traditional inflammatory indicators.^47^ The abnormal activation of IFN-induced signaling pathways is another critical factor that promotes the accumulation and function of inflammatory monocytes.^19^ Single-cell sequencing results showed that IFN-induced genes were significantly increased in monocytes derived from severely infected patients.^17,48^ CD169, a type I IFN-inducible receptor, is overexpressed in 93.7% of patients with COVID-19. CD169-positive monocytes are positively correlated with severe COVID-19 progression.^49^ CD169^+^ macrophages have been identified as a key subgroup of macrophages resident in lung tissue and significantly promoted the progression of inflammation.^50^ IFN-induced ACE2 expression in macrophages triggers inflammatory signals and CD147 in monocytes also participates in virus invasion and inflammation progression.^48^

Upregulation of the transcription factor MAFB inhibits the type I IFN response. The MAF transcription factor plays a vital role in regulating the production of pro-inflammatory macrophages induced by macrophage CSF (M-CSF). In malignant tumors, the silencing of MAF causes macrophages to reprogram to an M1-like phenotype and enhance anti-tumor activity. More importantly, MAFB interferes with the expression of IFN-1 pathway-related genes by regulating the activity of IRF3,^51^ which plays a key role in the disease progression of COVID-19 patients. The overexpression of MAFB and silencing of MAF are positively related to COVID-19 progression.^52^ Reversing the expression of these two transcription factors inhibits macrophage function and reduces the progression of pulmonary fibrosis. Therefore, MAFB and MAF have become accurate diagnostic indicators for estimating the progress of COVID-19. These two transcription factors are expected to become new therapeutic targets for blocking the progression of COVID-19.

### Recruitment

**Fig. 2:**
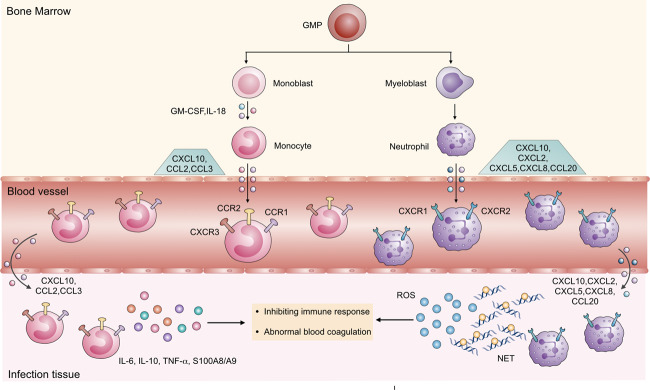
The differentiation and recruitment of monocytes and neutrophils in COVID-19. The myeloblasts differentiate into neutrophils in the bone marrow and are then recruited by chemokines such as CXCL10, CXCL2, CXCL5, CXCL8, and CCL20 to the peripheral blood and infected tissues. Pro-monocytes differentiate into monocytes, which migrate into the peripheral blood and infected tissues by binding chemokines such as CXCL10, CCL2, and CCL3. In infected tissues, IL-6, IL-10, TNF-α, S100A8/A9, ROS, and NETs derived from neutrophils or monocytes were increased and inhibited the immune response to induce pneumonia and lead to blood coagulation disorder

Chemokines recruit various immune or non-immune cells under pathophysiological conditions such as inflammation and tumors.^30^ The chemokine–chemokine receptor axis is the primary mechanism for the recruitment of monocytes and neutrophils in COVID-19. The release of chemokines CCL2, CCL3, and CXCL10 increased significantly in serum, leading to a significant increase in abnormally differentiated monocytes.^2,53^ In addition, the secretion of various inflammatory factors such as granulocyte-macrophage CSF (GM-CSF), IL-18, CCL2, CXCL10, and osteopontin in plasma increased to induce abnormal differentiation of myeloid cells.^15,54^ In the alveolar lavage fluid of severe COVID-19 patients, large quantities of chemokines CCL2 and CCL7 accumulate to recruit CCR2^+^ monocytes and promote local tissue inflammation.^55^ Lung tissues infected with COVID-19 secrete CXCL1, CXCL2, CXCL3, CXCL5, CXCL8, and CCL20 to recruit neutrophils by combining with CXCR1 and CXCR2^56^ (Fig. 2). Therefore, blocking the migration of these abnormal myeloid cells and reducing the aggregation of immature monocytes or neutrophils are essential means of developing targeted myeloid cells to treat COVID-19.

## Developing severe symptoms due to myeloid cells

Accumulation of abnormal myeloid cells promotes multiple organ damage through the secretion of abnormal cytokines, increased reactive oxygen species (ROS) production, and abnormal coagulation function, leading to the death of severe COVID-19 patients.

### Cytokines

The production of “cytokine storm” in CAR-T cell therapy for tumors has been verified to be mediated by monocytes.^57,58^ Infection with SARS-CoV-2, the accumulation of pro-inflammatory monocytes leads to the production of a “cytokine storm,” which aggravates the organizational damage of lung tissue. The results of single-cell sequencing showed that monocytes with high expression of TNF, IL-10, CCL3, IL-6, and other pro-inflammatory factors accounted for 98.3% of the monocytes in the peripheral blood of patients with severe infection (Fig. 2). The proportion of this group in the remission phase was reduced to 12.1% and was not analyzed in the peripheral blood of healthy controls.^59^ Another analysis showed that the secretion of IL-6 was strongly associated with sepsis.^60^ In terms of mechanism, this group of monocytes was detected with high expression of ATF3, NFIL3, and HIVEP2 transcription factors, suggesting that these three transcription factors were involved in the cytokine storm induced by monocytes. In the peripheral blood of severely infected patients, IL-6, IL-10, and TNF accumulate to cause cytokine syndrome. These cytokines are significantly negatively correlated with the aggression of T cells. IL-β has been shown to decrease in COVID-19-associated monocytes of infected patients compared to those of healthy individuals.

### NETs in neutrophil

The process by which neutrophils exert resistance to infection relies on neutrophil extracellular trap (NET) production. NETs are large extracellular reticulation structures composed of intracellular DNA or mitochondrial DNA in concert with intracytoplasmic components and granule proteins. NETs are initiated by NETosis, a specific form of neutrophil death. Neutrophils exert protection against infection processes mainly through entrapping, neutralizing, and killing microorganisms, such as bacteria and fungi, by NETs. The role of NETs in tumor progression and inflammatory responses is gradually being elucidated. Cytokines released by tumor cells such as G-CSF, CXCL8, and related chemokines have been shown to induce the production of NETs, which leads to immune escape of tumor cells and promote tumor growth and invasion, thus surrounding them and preventing them from being killed by immune cells.^61,62^

NETs are considered to be essential mediators inducing tissue damage in kinds of diseases. Overproduction of NETs by neutrophils induces lung tissue damage by combining neutrophil elastase and myeloperoxidase.^63,64^ On the other hand, NETs produced by neutrophils are significantly increased in severely infected patients. By interfering with the coagulation process regulated by normal platelets and thrombin, they cause thrombotic microangiopathy and accelerate the death of severely ill patients.^65^ Another study on neutrophils in COVID-19 patients showed that the number of neutrophils gradually increased as the disease progressed. RNA-sequencing results showed that neutrophils derived from patients were over-activated and 17 NET formation-related genes were significantly elevated.^63^ By analysing the lung specimens from death samples of SARS-CoV-2 infection and COVID-19 unrelated causes, it was found that the airway chambers and lung interstitium of each COVID-19 patient were rich in neutrophils and NETs, indicating that NETs may be a key factor driving severe lung complications.^66^

### ROS production

Bacterial infection upregulates the expression of factors related to clearing function, including the synthesis of granule protease, NADPH oxidase complex, phagocytosis, and chemotaxis.^67^ Similarly, in neutrophils infected with SARS-CoV-2, functional molecules required for clearing bacteria continue to exist. Oxidative stress is another mechanism by which neutrophils induce lung tissue damage. Oxidative stress results from an imbalance between oxidants and antioxidant agents, resulting in lipid peroxidation and DNA oxidation. In susceptible individuals, neutrophils migrate rapidly to target tissues and release excessive ROS. ROS-induced lung tissue and red blood cell functional damage are regarded as the main factors inducing hypoxic respiratory failure in severe COVID-19.^68^ Previous studies have suggested that neutrophils migrate from the vascular lumen to tissues in one direction. Recently, other studies have shown that neutrophils migrate in reverse to the bloodstream. This process is the reverse transendothelial migration of neutrophils. The characteristics of these neutrophils prolong their passage through capillaries, leading to multiple organ failure.^68^ In addition, the production of excessive reactive oxygen leads to the oxidation of unsaturated fatty acids in the cell membrane, which affects oxygen and carbon dioxide diffusion and the deformability of red blood cells in the capillaries, increasing platelets.

Moreover, such modification of red blood cell membranes can cause reactivation of neutrophils, affect the release of ATP and NO, and cause abnormal oxygen transport and vasodilation. However, excessive production of ROS leads to an imbalance of Fe^2+^ and Fe^3+^. It is well known that only Fe^2+^ can be combined with oxygen; however, ROS oxidize a large amount of Fe^2+^ to Fe^3+^, which decreases the oxygen-binding rate and, therefore, oxygen transport; this causes the body to enter an anoxic state and accelerates multiple organ failure.

### Abnormal blood coagulation

In severe COVID-19 patients, abnormal blood coagulation caused by abnormal differentiation of myeloid cells is another leading cause of patient death. Over-activated monocytes and neutrophils promote inflammation, leading to histopathological damage, and induce coagulation dysfunction in the body. Viruses attack vascular endothelial cells and cause damage, and activated monocytes are recruited to the infected tissues. Monocyte-derived microvesicles and endothelial cells activate exogenous blood coagulation pathways, resulting in fibrinogen deposition and blood clotting. NETs released by neutrophils trigger the blood coagulation contact pathway, bind and activate platelets, and enhance blood coagulation.

### Supplementary functions

In addition to the direct inhibitory effect on T cells, neutrophils accelerate Th17 cell differentiation from naive CD4 T cells, leading to the death of critically ill patients. It has been reported that Th17 cells promote the progression of inflammation in many diseases and they represent another critical neutrophil-mediated mechanism that aggravates the progression of pneumonia.^69^

In COVID-19 patients, the classic metabolism of monocytes is destroyed. In patients with severe COVID-19 pneumonia, the corpuscle trajectory of ATP-purinergic signal transduction in monocytes is blocked, which is an important mechanism leading to the progression of pulmonary fibrosis.^23^ Another study on organelles showed that monocytes derived from the peripheral blood of patients exhibit mitochondrial dysfunction and aerobic glycolysis has been reduced.^54^

## Monocyte in age, sex, or diabetes

Elderly patients infected with COVID-19 have a more pronounced inflammatory response and worse prognosis than younger patients.^70^ Compared with younger patients, the phagocytic function or HLA-DR expression in monocyte was impaired. Moreover, the inflammatory factors derived from monocytes significantly increased in elderly patients. Mechanistic research shows that the abnormal characteristics of monocytes in elderly patients may be related to mitochondrial dysfunction in senescent monocytes.^54^ In addition, male patients infected with COVID-19 have more severe symptoms and suffered to the higher mortality rate than female patients because of the increased accumulation of non-classical monocytes in the peripheral blood and more secretions of IL-8 and IL-18.^71^ These monocytes reduced T-cell function to cause severe symptoms in male patients. In addition, the reasons why diabetic patients become critically ill after being infected with COVID-19 has also been studied. Among them, after receiving high glucose signals, HIF-1α induces monocytes to increase glycolysis and produces more immunosuppressive cytokines to reduce T-cell activity.^72^ This provides evidence to link the severity of COVID-19 to diabetes. These differences would provide the individualization therapy of targeting myeloid cells.

## Targeting therapy from myeloid cells

Single-cell sequencing analysis revealed that myeloid cells had abnormal differentiation trajectories in the circulation of infected lung tissue of COVID-19 patients. The accumulation of these immature monocytes and granulocytes causes lung tissue damage to promote the adverse prognosis of COVID-19 through the secretion of cytokines or direct inhibition of T-cell functions. Therefore, neutralizing the cytokines produced abnormally by myeloid cells or targeting checkpoints that lead to abnormal differentiation and dysfunction of myeloid cells has become the crucial approaches to block the occurrence of severe COVID-19.

### Cytokine neutralization

**Fig. 3:**
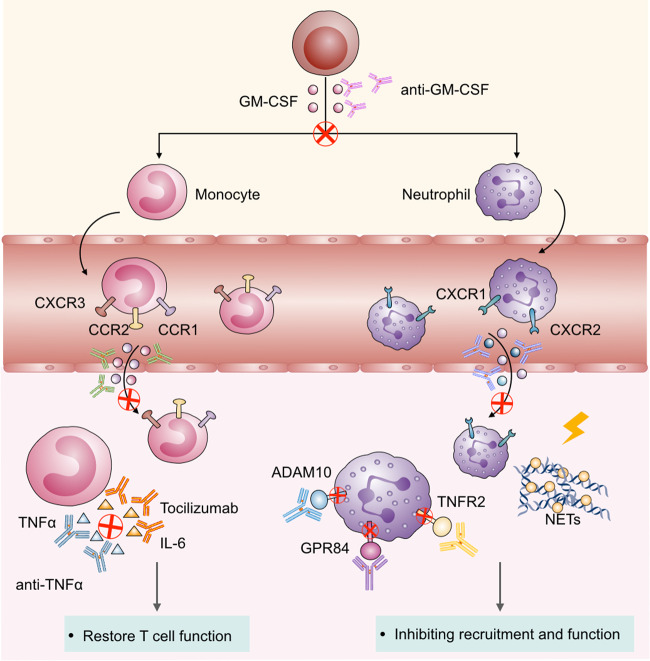
Therapeutic strategies targeting myeloid cells in COVID-19. Targeting GM-CSF blocks the differentiation of myeloid cells into neutrophils. Using chemokine antibodies could block the recruitment of monocytes and neutrophils into the infected tissue. Treatment with tocilizumab and anti-TNF-α, which blocks IL-6 and TNF-α secretion by monocytes, can restore T-cell function. Antibodies targeting checkpoints such as ADAM10, GPR84, and TNFR2, which are highly expressed on neutrophils, can inhibit the function of neutrophils. Targeting NETs is a therapeutic intervention for neutrophils in COVID-19

Multi-organ functional damage induced by “cytokine storm” has become a leading cause of death in severe COVID-19 patients.^73^ Neutralizing cytokines have become an important channel for the early exploration and treatment of critically ill patients. After tocilizumab treatment, the cytokine storm in patients entering the remission period was significantly suppressed and blocking the signaling pathway of IL-6, which is secreted by macrophages, would be clinically beneficial.^74^ A study from China showed that the clinical benefit rate was 75%, whereas a study from Italy showed it to be only 33%. TNF-α, another important factor secreted by myeloid cells, also contributes to lung fibrosis and multiple organ failure. Etanercept and infliximab, the TNF/TNF receptor blockers that have been approved for the therapy of rheumatoid arthritis, have achieved specific effects in clinical trials for severe COVID-19 patient treatment; however, there is still a massive gap between its ideal clinical effect (NCT04425538) (Fig. 3).

The blockade of GM-CSF reduces the inflammatory response by reducing neutrophil aggregation and clinical trials are ongoing. GM-CSF is a critical factor in inducing the differentiation of myeloid cells to neutrophils. It is significantly increased in most severe COVID-19 and is positively correlated with disease progression. Therefore, targeting GM-CSF is a potential target for the treatment of COVID-19-related inflammation.^53^ The process by which neutrophils induce Th17 cell aggregation depends on the presence of NOS; therefore, NOS neutralization can be used as another method to inhibit the progression of severely infected patients.^69^ In addition, reprogramming mesenchymal stem cells to secrete FGF7 and IGF1, and differentiate them into myeloid cells with anti-inflammatory and phagocytic abilities is another therapeutic strategy.^75^

Blocking recruitment is another approach that inhibits abnormal monocyte or neutrophil accumulation. There was an enrichment of chemokines such as CCL4L2, CCL3, and CCL4, and their respective receptors in severe stage monocytes, demonstrating that drugs targeting these cytokines and/or their receptors signaling pathway are potentially used to treat severely infected patients. CXCL8, also known as IL-8, which was first identified, has been studied in neutrophil recruitment. In malignant tumors, IL-8 high secretion is negatively correlated with the efficacy of immune checkpoint inhibitors.^76^ In patients with COVID-19, CXCL8 is highly secreted in the circulation and infected lung tissues. Blockers such as BMS-986253 may develop new ways to reduce neutrophil accumulation and alleviate tissue impairment in the clinic.^77^

We have summarized the related clinical trials to myeloid cells in Table 1, mostly targeting IL-6/IL-6R, GM-CSF, CXCR1/2, IL-1β, and TNF-α. However, the completed clinical trials show that the effects of these treatment schemes are quite different. During the treatment of IL-6 antagonist tocilizumab against COVID-19, it may lead to the pro-inflammatory effect of cells that do not express IL-6 receptor, resulting in ineffective disease treatment.^78^ Another clinical trial of tocilizumab showed a significant reduction of mortality in 28 day.^79^ Tofacitinib, as a JAK1 and JAK3 inhibitor, simultaneously regulates the effect of IL-6, reduces the release of cytokines from Th1 and Th17, and reduces the risk of death and respiratory failure.^80^ Canakinumab, an anti-IL-1β antibody, resulting 16% patients suffered with serious adverse events. In general, targeting IL-1β treatment did not significantly increase the possibility of not requiring invasive mechanical exploitation for 29 days.^81^ The reason for these differences might relate to the difference of cytokine secretion level. In mild patients, the level of IL-6 and TNF-α were increased, but the production of IL-1β is even lower than that in healthy people. This diverse cytokine spectrum of monocytes may be related to the acute hyperplasia of the bone marrow caused by COVID-19 infection. Therefore, the treatment of targeting myeloid cells needs to be individualized and after fully detecting the expression of related cytokines or receptors, a precise targeted treatment plan can be formulated to maximize the effect and improve the patient’s prognosis.

**Table 1:** Major clinical trials related to myeloid cells for treatment of COVID-19

Candidate therapeutic	Target	Phase	Registration number	Initiation time	Status	Principal investigators and their unit	Number of patients	References
Tocilizumab	IL-6	II	NCT04317092	Mar 19, 2020	Active, not recruiting	National Cancer Institute, Naples	402	
		III	NCT04356937	Apr 20, 2020	Completed	Massachusetts General Hospital	243	^88^
		IV	NCT04377750	Apr 8, 2020	Recruiting	Hadassah Medical Organization	500	
		II	NCT04750317	May 11, 2020	Completed	I.M. Sechenov First Moscow State Medical University	414	
		III	NCT05002517	Sep 3, 2020	Active, not recruiting	Asociacion Instituto Biodonostia	60	
		III	NCT04409262	June 16, 2020.	Completed	Hoffmann-La Roche	649	
		II	NCT04363736	May 5, 2020	Completed	Hoffmann-La Roche	97	
		II	NCT04445272	May 22, 2020	Completed	Fundacion SEIMC-GESIDA	495	
		III	NCT04320615	Apr 3, 2020	Completed	Hoffmann-La Roche	452	
Siltuximab	IL-6	II	NCT04329650	Apr 15, 2020	Recruiting	Judit Pich Martínez, Fundacion Clinic per a la Recerca Biomédica	200	
Mavrilimumab	GM-CSF	II	NCT04492514	May 20, 2020	Recruiting	Kristin Hudock	60	
		II	NCT04397497	May 22, 2020	Not yet recruiting	Ospedale San Raffaele	50	
		II	NCT04399980	May 20, 2020	Completed	The Cleveland Clinic	40	^89^
		II	NCT04463004	Sep 2, 2020	Completed	Virginia Commonwealth University	2	^89^
		II	NCT04447469	Jul 27, 2020	Recruiting	Kiniksa Pharmaceuticals, Ltd.	588	
Lenzilumab	GM-CSF	II	NCT04583969	Oct 19, 2020	Recruiting	National Institute of Allergy and Infectious Diseases	400	
		III	NCT04534725	Dec 17, 2020	Recruiting	Peter MacCallum Cancer Centre, Australia	2282	
Gimsilumab	GM-CSF	II	NCT04351243	Apr 12, 2020	Completed	Kinevant Sciences GmbH	227	
Tofacitinib	GM-CSF	II	NCT04750317	May 11, 2020	Completed	I.M. Sechenov First Moscow State Medical University	414	
Baricitinib	JAK	III	NCT04970719	Jul 10, 2020	Recruiting	Wasim Md Mohosin Ul Haque, Bangladesh Institute of Research and Rehabilitation in Diabetes, Endocrine and Metabolic Disorders	382	
		II	NCT04321993	Apr 17, 2020	Recruiting	Lisa Barrett, Nova Scotia Health Authority	800	
		III	NCT04401579	May 8, 2020	Completed	National Institute of Allergy and Infectious Diseases (NIAID)	1033	^90–92^
		III	NCT04421027	Jun 12, 2020	Completed	Eli Lilly and Company	1585	^93^
Reparixin	CXCR1/2	II	NCT04794803	May 5, 2020	Terminated	Dompé Farmaceutici S.p.A	55	
		III	NCT04878055	Feb 14, 2021	Recruiting	Dompé Farmaceutici S.p.A	312	
Canakinumab	IL-1β	III	NCT04362813	Apr 30, 2020	Completed	Novartis Pharmaceuticals	454	^83^^,^^94^
		II	NCT04365153	Apr 24, 2020	Completed	The Cleveland Clinic	45	
Infliximab	TNF-α	III	NCT04593940	Oct 15, 2020	Recruiting	Daniel Benjamin, Duke University	2160	
		II	NCT04425538	June 1, 2020	Completed	National Institutes of Health (NIH)	17	

### Checkpoints

In addition to the elevated expression of chemokine receptors CXCR1 and CXCR2, the neutrophils gathered in the infected lung tissue also showed increased expression of the inflammatory proteins GPR84 and ADAM10. GPR84 is a member of the orphan receptor in the G Protein-Coupled Receptor (GPCR) family and was first identified in neutrophils stimulated by GM-CSF.^82^ GPR84 has previously been reported to be a critical regulatory molecule that promotes the release of inflammatory factors.^83^ Its blocker GLPG-1205 reduces dextran sodium sulfate-induced enteritis and has been used in phase 2 clinical trials.^84^ PBI-4050, another A GPR84 antagonist for diabetic nephropathy or lung fibrosis treatment, has entered phase 2 clinical trials.^85^ Therefore, targeting GPR84 to inhibit the infiltration and function of neutrophils may be another effective means of organizing COVID-19 lung tissue fibrosis. Another inhibitor of ADAM10 has undergone preclinical studies to analyze its effectiveness in inhibiting the release of inflammatory factors and pulmonary fibrosis. Aminopeptidase-N is a possible coronavirus receptor and neutrophil-specific gene, which may be blocked by the approved drug ezetimibe. Moreover, emerging experimental evidence also showed that it interacts with tosedostat and ubenimex.^56^

Furthermore, recent studies have found that the combined application of oseltamivir and CXCR2 antagonists reduce neutrophil recruitment in the lung tissue of mice infected with the influenza virus.^86^ In addition, the usage of hydroxychloroquine, azithromycin, and colchicine for COVID-19 therapy is being investigated because of their ability to block neutrophils. Similarly, blockade of CCR2 or CCR5 to reduce immature monocytes migrating into inflammatory tissues is essential to reverse the damage to lung tissue. NETs can cause inflammation-related lung injury, thrombosis, and fibrosis, suggesting that NETs may be targeted for therapeutic intervention.^87^

## Conclusion

Although single-cell sequencing exhaustively describes the differentiation trajectory of myeloid cells in the peripheral blood and infected lung tissue of patients with COVID-19, the accumulation of abnormal monocytes and neutrophil subpopulations is a critical factor in the prognosis of critically ill patients. The mechanisms underlying the accumulation of these abnormal myeloid cells require further research. Moreover, cytokines such as IL-6 are the critical factors for these abnormal myeloid cell subgroups to promote disease progression; however, targeting a specific cytokine or chemokine receptor has not achieved an ideal therapeutic effect. Therefore, the discovery of specific targets for COVID-19 and the development of combined treatment strategies are the next steps in targeting myeloid cells to reverse severe COVID-19.
